# Deltamethrin-Induced Respiratory and Behavioral Effects and Adverse Outcome Pathways (AOP) in Short-Term Exposed Mozambique Tilapia, *Oreochromis mossambicus*

**DOI:** 10.3390/toxics10110701

**Published:** 2022-11-17

**Authors:** Azubuike V. Chukwuka, Shubhajit Saha, Dip Mukherjee, Priyajit Banerjee, Kishore Dhara, Nimai Chandra Saha

**Affiliations:** 1 National Environmental Standards and Regulations Enforcement Agency (NESREA), Osogbo 234, Osun State, Nigeria; 2 Department of Zoology, Sundarban Hazi Desarat College, Canning 743611, West Bengal, India; 3 Department of Zoology, S.B.S. Government College, Hili 733126, West Bengal, India; 4 Fisheries Ecotoxicology Research Laboratory, Department of Zoology, University of Burdwan, Bardhhaman 713104, West Bengal, India; 5 Directorate of Fisheries, Government of West Bengal, Kolkata 700091, West Bengal, India

**Keywords:** structural homology, in silico analysis, neurotoxicity, behavioral toxicity, respiratory distress

## Abstract

Disrupted behavior and respiratory distress effects of 96-h acute deltamethrin exposures in adult Mozambique tilapia, *Oreochromis mossambicus*, were investigated using behavioral indices and opercular movement, respectively. Deltamethrin concentrations were found to be associated with toxicological (lethal and sublethal) responses. At 24, 48, 72, and 96 h, the LC50 values and 95% confidence limits were 12.290 (11.174–14.411 µg/L), 12.671 (11.334–15.649 µg/L), 10.172 (9.310–11.193 µg/L), and 8.639 (7.860–9.417 µg/L), respectively. The GUTS-model analysis showed that GUTS-SD (stochastic death) with a narrow tolerance distribution in deltamethrin exposed *O. mossambicus* populations was more sensitive than the GUTS-IT (individual tolerance) model. Prior to death, exposed fish demonstrated concentration-dependent mortality and disturbed behavioral responses, including uncoordinated swim motions, increased mucus secretion, unbalanced and unpredictable swimming patterns, and inactivity. The altered behavioral patterns and increased opercular movement with increased deltamethrin levels and exposure time are strongly suggestive of neurotoxicity and respiratory distress, respectively. Adverse Outcome Pathways (AOPs), describing biological mechanisms and plausible pathways, highlighted oxidative stress and cholinergic effects as intermediate steps linked to respiratory distress and behavioral toxicity.

## 1. Introduction

The increasing demand for pesticides and fertilizers, alongside the rising demand for crops, goods, and services, is a recurrent concern for sustainability and environmental stewardship [1]. While pesticides and fertilizers do provide benefits, their production and use incur costs, including acute and long-term ecological health effects [2]. The sustainable development goal report articulated the need to improve the understanding of current practices and drivers of pesticide and fertilizer use, and identify knowledge gaps regarding environmental and health risks [3]. Due to current management practices, legislation, and policies to minimize adverse environmental and health impacts, SDGs specify that the environmental footprint of crop protection should be minimized [4].

Pyrethroid insecticides are preferred over organochlorine and organophosphate insecticides due to their powerful insecticidal properties and low toxicity to most non-target animals, particularly mammals. However, some are highly harmful to all aquatic creatures [5,6]. In particular, fish are the most vulnerable class of nontarget aquatic species due to their enzyme-system deficiency in hydrolyzing pyrethroids [7]. Although the larvicidal regimen of pyrethroids in aquatic systems increases risks and potential hazards to fish [7], deltamethrin, one of the most commonly used, has been linked to a wide number of stress-related outcomes in fish [8]. 

Classed as a moderately toxic substance (WHO Class II), deltamethrin induces neurotoxicity in insects and imminent death via paralysis by modifying the kinetics of voltage-sensitive sodium channels and increasing nerve membrane sodium permeability [9,10]. The greater susceptibility of fish to its effects compared to other endotherms, such as birds and mammals, is attributable in part to their lower metabolic rate and inefficient pesticide metabolic breakdown [11]. Several investigations have indicated that deltamethrin toxicity in fish manifests as neurotoxicity (i.e., blocking sodium channels, decreasing acetylcholinesterase activity), oxidative stress, impairment of cellular immunity, endocrine disruption, and inhibiting gamma-aminobutyric acid receptors [12,13,14]. Impacts on the gills and liver have also been documented [11,15]. This implies that, beyond the behavioral effects derivable from neurotoxicity [16,17], respiratory disruption is a plausible effect attributable to gill toxicity [18].

Pyrethroids selectivity for aquatic vertebrates and the marked susceptibility of fish is explainable on the basis of toxicokinetic (i.e., absorption, biotransformation, distribution, elimination) and toxicodynamic (i.e., biochemical/physiological effects) factors [19]. Documented environmental concentrations of deltamethrin range from 1.8 to 253.0 ng L^−1^ in freshwater [20]. Its toxicity to freshwater life is estimated to range from 0.0001 μg L^−1^ (48- and 96-h EC_0_ for immobilization of *Daphnia magna*) to 0.75 μg L^−1^ (for oxidative stress in catfish under 48 h exposures) [21,22]. Since the sensitivity of fish to various pesticides is species-specific and niche-specific [18,23], assessing species-specific exposures will provide information on the risk spectrum of specific pesticides. Furthermore, the lower lipophilicity (moderate aqueous solubility) (log Kow = 4.6) and lower bioavailability of deltamethrin in the presence of suspended sediment [24,25] indicates that pelagic species have a greater risk likelihood than benthic species. Tilapias particularly occur under a wide range of geographic and climatic conditions, with *Oreochromis * spp. notably occupying a pelagic niche [26,27]. While the short life of pyrethroids in most vertebrates necessitates acute exposure investigations [28], altered gill pathology in sub-acute deltamethrin-exposed tilapia [29] also raises concerns about impaired respiration in the wild. Thus, we evaluated respiratory distress and behavioral alterations in *Oreochromis mossambicus* under acute deltamethrin exposure regimes. 

To unravel mechanistic possibilities and affinities with biomolecules, the chemical structure of single chemicals has been subjected to extensive computational investigations and homology modeling [30]. These in silico (computational simulation or modeling) methods are useful in indicating the presence or absence of a toxic property [31]. More reports have indicated that these models could complement or eventually replace animal testing [31,32]. Furthermore, to provide information and visualize possible mechanistic pathways for deltamethrin toxicity to fish respiration and behavior, we attempted to develop the Adverse Outcome Pathway (AOP) framework using evidence-based information. Aside from seeking to establish the median 50%, 40%, 30%, 20%, and 10% hazardous concentrations (LC_50_, LC_40_, LC_30_, LC_20_, and LC_10_), this study specifically aimed to evaluate the acute toxicity of deltamethrin to Mozambique tilapia, *Oreochromis mossambicus*, using respiratory distress and behavioral toxicity as endpoints.

## 2. Materials and Methods

### 2.1. Experimental Procedures

The proper quality assurance methods of sample preparation, handling, and preservation were used in accordance with US EPA guidelines [33]. Adult *Oreochromis mossambicus* (average length 52.50 ± 3.26 mm, average weight 23.70 ± 5.49 g; n = 120) were used as test organisms for this study. The stock was obtained from a local fish culture facility. Fish acquired for this study were acclimatized for 7 days before the experiment started. During the process, they were kept in concrete vats of 1000 L volume of chlorine-free tap water (pH 7.10 ± 0.45; temperature 28.63 ± 1.54 °C) under a 12-h photoperiod. The fish were fed a commercial pellet diet made by AQUAmunch^®^ African pellets with 30% crude protein, 35% carbs, and 8% lipids.

Deltamethrin (25% EC), a technical grade broad spectrum insecticide (CAS No. 52918-63-5; C_22_H_19_Br_2_NO_3_) manufactured by Bayer Crop Science Ltd., Gujarat, India, belonging to the pyrethroid class of insecticide, was used for evaluation of its toxicity. Using stocking density of 5g of fish to 1L of water [34], static renewal bioassays were performed on healthy Mozambique tilapia in 60 L glass aquaria with 47 L of unchlorinated tap water. Physicochemical properties of the test medium were monitored and maintained as follows: temperature 28.5 ± 2.25 °C; pH 7.8 ± 1.50; free CO_2_ 10.9 ± 1.50 mg/L; DO 5.90 ± 0.75 mg/L; alkalinity 174 ± 9.60 mg/L as CaCO_3_; hardness 119 ± 4.50 mg/L as CaCO_3_.

Static-renewal bioassay exposures were carried out in quadruplicates per treatment, and the control (*n* = 10 per tank). The fish were not fed for 24 h prior to the start of the bioassay. The ratio of toxicant to water in each tank was modified according to predetermined exposure concentrations. An initial 24-h rough range finding test was performed to determine the dose range at which fish death occurs [35]. Ten exposure concentrations (5.0, 6.0, 7.0, 8.0, 9.0, 10.0, 11.0, 12.0, 13.0, and 14.0 µg/L) of deltamethrin and control group were used to determine the 24–96-h median lethal concentration (LC_50_). Nominal values were used due to the difference between nominal and observed concentrations; i.e., <5 %. To avoid deteriorating water quality from the buildup of organic deposits in the aquaria, dead fish were immediately removed. Mortalities per tank were documented every 24 h. Toxicant concentration in exposure media was ensured by siphoning 10% of the test water and replacing it with the same percentage of freshly prepared test solution every 24 h [36,37].

### 2.2. Sublethal Toxicity Endpoints

#### 2.2.1. Behavioral Toxicity

During the bioassay, physical observation revealed ethological responses such as irregular swimming, mucus discharge, and loss of balance in the exposed fish [16]. Behavior endpoints such as erratic swimming (jerking or irregular swimming patterns and fast swimming), mucus secretion (copious quantity visibly dripping over the body or gills), and loss of balance (predominant orientation of body tilted on the dorsal side or resting on one side of the body) were documented in the test organisms that were exposed to various concentrations of the toxicant for the 96-h test. During the bioassay, responses in the fish were measured by physical observation [38]. All of the behavioral characteristics of the treated fish over those of the control group were recorded as the number of individuals/24-h interval. The results for each concentration were averaged over all the replicates, and the percentage values were graphically presented [39].

#### 2.2.2. Respiratory Distress

The oxygen level in each control and treatment tank was measured after 24, 48, 72, and 96 h of exposure. Winkler’s iodometric method was used to calculate how much oxygen was used by each exposure group during the intervals of the 96-h experiment [17,40]. The oxygen consumption rate of the fish exposed to the toxicant was computed and graphed as a mean mg/h/g body weight/L after the experiment.

### 2.3. AOP Development

AOPs organize knowledge regarding the course of toxicity via biological levels of organization. AOPs lay the groundwork for mechanism-based alternative testing techniques to hazard assessment by determining the links between toxicity events at different levels. The pathway chosen in this investigation was based on sublethal endpoints at several levels of biological organization, as indicated by previous research using deltamethrin-specific studies in fish. In the absence of deltamethrin-specific studies, evidence-based research for chemicals homologous to deltamethrin was used to develop plausible pathways for behavioral and respiratory toxicity [41].

To improve the accuracy of the AOP using homologous chemicals, in silico analysis of the tertiary structure of deltamethrin was carried out against a chemical database to highlight existing chemicals with close similarity based on conserved functional groups. Morgan fingerprints and GenRA analysis were achieved using the EPA CompTox Chemicals dashboard and the U.S. EPA’s Toxicity Reference Database (ToxRefDB) version 2.0 [42].

### 2.4. Statistical Analysis

The mortality rates of *O. mossambicus* at various deltamethrin concentrations and exposure times were analyzed using R software program 2.14.0 USEPA [33] and Finney’s probit assessment [43] for determining lethal concentrations (LC_50_) for the period (24, 48, 72, and 96 h) with 95% confidence limits. At 96 h, toxicological endpoints such as LOEC (Lowest Observed Effect Concentration) and NOEC (No Observed Effect Concentration) were calculated using acute toxicity data. MATC (Maximum Allowed Toxicant Concentration) was calculated by multiplying the 96-h LC_50_ result by Application Factor 0.1 [44]. Toxicity factors (TF) for *O. mossambicus* were calculated using the technique (TF = LC_50_ at 24 h/LC_50_ at any other exposure time) based on median lethal concentrations [45]. Using the OpenGUTS^®^ program, the GUTS model was used to determine the survival patterns of *O. mossambicus* when they were exposed to deltamethrin during a 96-h exposure. The parameters entered into the model computation included kd (the dominant rate constant), MW (the threshold distribution’s median), HB (the background hazard rate), and bw (the killing rate, which is exclusively needed for SD) [46]. 

Mean differences in observed behavioral and respiratory distress indices were analyzed using one-way ANOVA. Correlation analysis was used to associate behavioral indices, respiratory index, and mortality across exposure concentrations. The statistical significance level was set at *p* < 0.05. Bar charts were created using GraphPad Prism (Version 9.1.2). The Kaplan-Meier estimator was used to calculate fish survival rates during the exposure time. Correlation analysis was conducted using OriginPro (Version 2022b).

## 3. Results and Discussion

### 3.1. 96-h Acute Toxicity Studies

**Figure 1:**
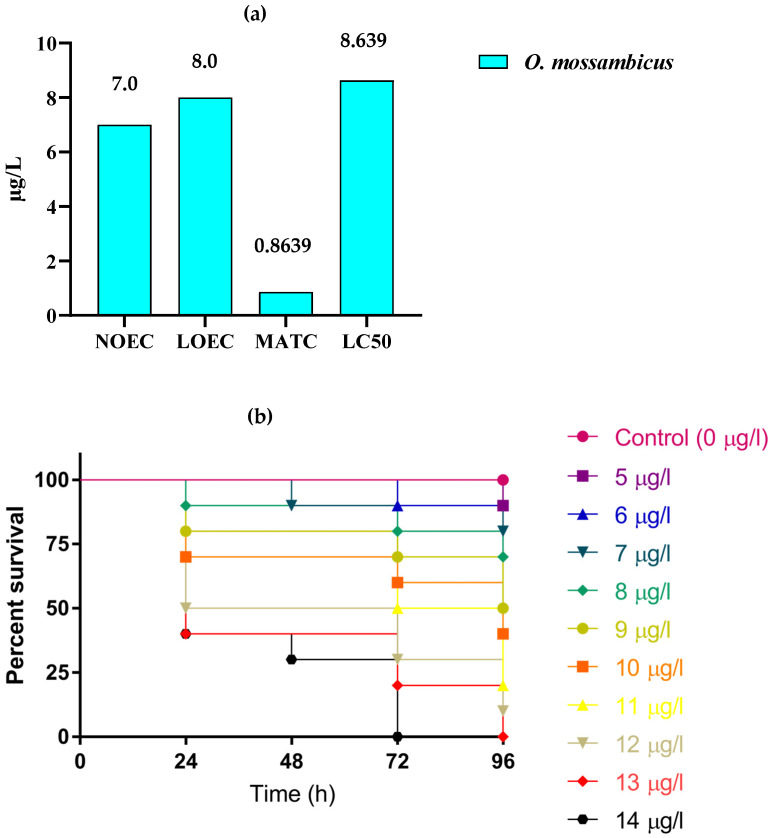
(**a**) Figure 1. NOEC and LOEC (after 24 h); MATC and LC_50_ value (after 96 h) for *O. mossambicus* exposed to deltamethrin. (**b**) Kaplan–Meier survival curves of *O. mossambicus* exposed to various exposure concentrations of deltamethrin (log-rank (Mantel-Cox) test: Chi square-88.14; df 10; *p* value: < 0.0001).

Table 1 shows the deltamethrin lethal concentrations for *O. mossambicus* over a 24–96-h period (LC_10, 20, 50_) and the 95% fiducial limits. In the control group, none of the test animals died during the test. There was a high correlation between the number of deaths in the test animals and how long they were exposed to the chemicals (*p* < 0.05). Figure 1a summarizes the NOEC, LOEC, MATC, and 96-h LC_50_ values for *O. mossambicus* at 96 h.

The Kaplan-Meier curve shows that the overall survival rates of *O. mossambicus* were lower in the deltamethrin group than in the control group (Mantel log-rank test; *p* < 0.05) depending on the concentration and duration of exposure (Figure 1b). The chi square value for *O. mossambicus* was 88.14, the df was 10, and *p* < 0.0001. *O. mossambicus* survival rate decreased as deltamethrin concentrations and exposure durations (24, 48, 72, and 96 h) increased.

### 3.2. General Unified Threshold Model of Survival (GUTS) Analysis

**Figure 2:**
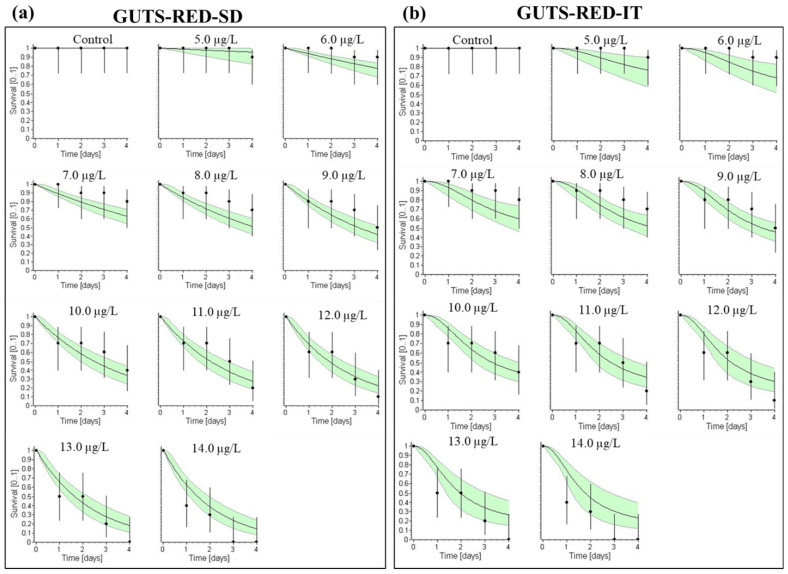
Relative fit of observed and fitted values of the (**a**) GUTS-SD and (**b**) IT models at different deltamethrin exposure concentrations.

Figure 2 depicts the GUTS model fit where the tolerance or survival distribution was narrow for the GUTS-SD compared to the GUTS-IT which showed wider tolerance/survival distribution. Simulations using the GUTS-SD model revealed a well-fitted survival rate at 0.0 µg/L for all concentrations of deltamethrin. The survival rate at 0 mg/L for all concentrations of deltamethrin in the GUTS-IT model show a good fit. For *O. mossambicus*, the survival rate fits well at 0.0 µg/L at all exposure concentrations of deltamethrin for the GUTS-SD model simulation, but is overestimated at 14.0 µg/L, and underestimated for 7.0 and 8.0 µg/L (Figure 2a). Furthermore, for the GUTS-IT simulation analysis, the rate of survival precisely fits at 0.0 µg/L for all concentrations of deltamethrin, but is overestimated at 14.0 µg/L and underestimated for 5.0, 6.0 and 7.0 µg/L. Furthermore, based on AIC values, the fitted performance of GUTS–SD (249.03) in fish is better than that of GUTS–IT (271.46) (Figure 2b). At the acute level, the GUTS-SD model outperforms the GUTS-IT model in predicting the survival rate observed in *O. mossambicus* following exposure to deltamethrin. The greater predictive value of the GUTS-SD model compared to the GITS-IT strongly indicates that the response of exposed fish to deltamethrin exposure will elicit a shared threshold above which the exposed population will experience random deaths (stochastic death). Toxicodynamic inferences from the stochastic death model inherently indicate that since the survival threshold is shared by the population, toxicity responses will more likely elicit systematically faster compensation and damage repair mechanisms than if individual tolerance was applicable [47]. This faster recovery potential predicted by the stochastic model has been attributed to the narrow tolerance distribution in the test population [47]. Toxicodynamic recovery encapsulates cellular and physiological compensating mechanisms and repair that underly an organism’s stress response to chemical insult [48].

### 3.3. Toxicity Factor and Safe Level Assessment

The toxicity factor values for *O. mossambicus* exposed to deltamethrin for various exposure durations were 1.00, 0.970, 1.218, and 1.423 for 24, 48, 72, and 96 h, respectively. The magnitude of the deltamethrin toxicity factor for the target organism progressively increased with time. Acute toxicity is caused by a combination of physical, chemical, and biological events [49]. The LC_50_ value measures a population’s tolerance to a pollutant and is an effective tool for toxicity testing [50]. Tolerance is an important stage in organisms’ adaptation to hostile conditions [51]. It gradually rises over time due to decreased absorption, higher excretion, or variable degrees of metal transfer to less sensitive target areas [52]. According to Hart, Weston [53], estimated safe level of deltamethrin for *O. mossambicus* was 0.4041 µg/L.

### 3.4. Respiratory Distress

**Figure 3:**
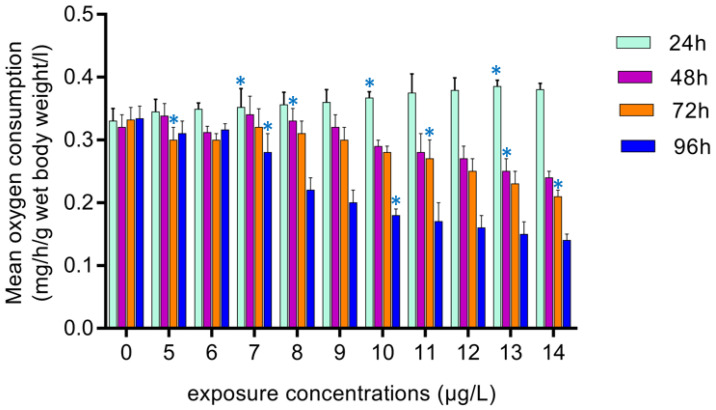
Mean oxygen consumption of *O. mossambicus* (mg/h/g wet body weight/L) exposed to various exposure concentrations of deltamethrin during various exposure periods. Asterix indicates significant differences in mean parameters (*p* < 0.05) across exposure intervals for each concentration.

Figure 3 illustrates the mean oxygen consumption of *O. mossambicus* exposed to deltamethrin versus control over a range of exposure times (24–96 h). The rate of mean oxygen consumption of *O. mossambicus* decreased with increasing exposure concentrations at various durations of exposure when compared to the control.

### 3.5. Behavioral Toxicity

**Figure 4:**
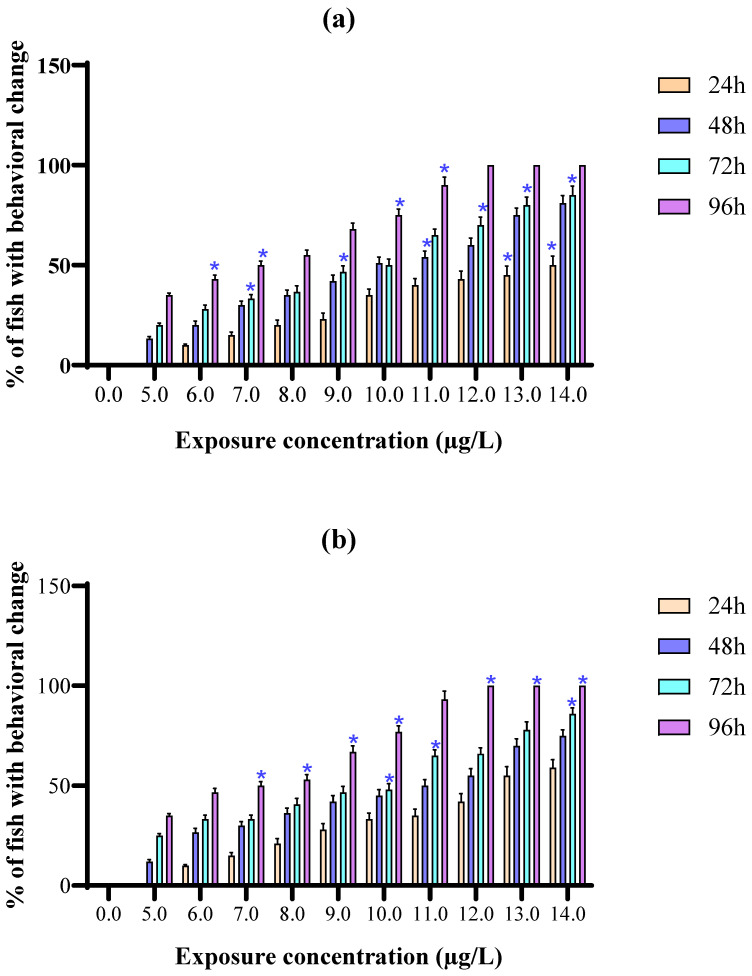

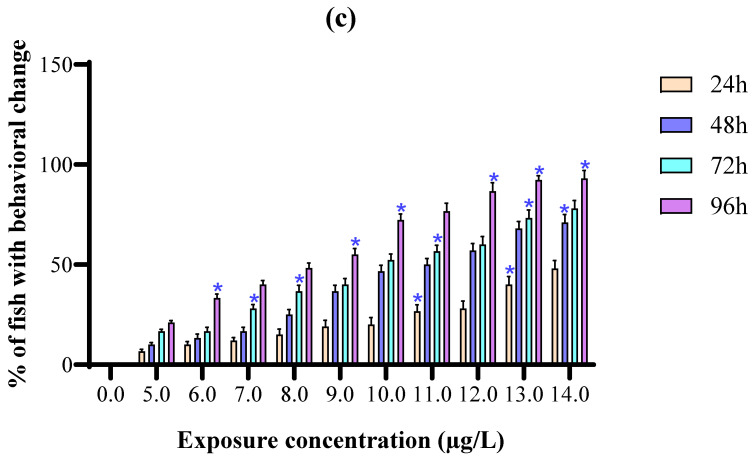
Changes of behavioral parameters (**a**) mucous secretion, (**b**) erratic swimming behavior, and (**c**) loss of balance behavior in *O. mossambicus* exposed to different concentrations of deltamethrin. Asterix indicates significant differences in mean parameters (*p* < 0.05) across exposure intervals for each concentration.

The present study’s observation of the ethological responses of the fish (Figure 4a–c) could serve as a useful indicator of the toxicity of deltamethrin to the ecosystem. After 96 h of exposure, *O. mossambicus* showed signs of stress, such as irregular swimming, mucous discharge, and loss of balance, as time and concentration increased, particularly at higher concentrations (12.0, 13.0, and 14.0 µg/L) (Figure 4a–c).

Somersaulting by fish was seen at higher levels. This was perhaps a first sign of their avoidance response to the test substance [54]. At first, the treated fish appeared to be more hyperactive than the control fish. *O. mossambicus* began to show signs of stress buildup with the progression of time and a gradual increase in concentration, such as erratic swimming, restlessness, gasping for air, and surface adhesion. Additionally, at the upper dose level, fish were also seen to exhibit a somersaulting motion. This behavior most likely represented a deltamethrin escape response [55]. *O. mossambicus* produces a lot of mucus as a defense mechanism to prevent deltamethrin from entering the body. It is a result of stress and has an unpleasant impact that is comparable to many other neurotoxins. Wheezing, repeated turning of the opercula, loss of balance, disturbance in buoyant behavior, increased rigidity, and a brief cessation of respiration were all signs of fish death at all exposures. The detrimental effects of the pyrethroid class of the pesticide, deltamethrin, on the cerebrospinal neural system were likely to blame for the restlessness and irregular swimming of treated fish [56,57]. The discrepancies in ethological reactions in the treated fish were most likely caused by internal abnormalities of the physiological functions, such as inhibition of enzyme activities, impairment in neuronal transmission, and malfunctions in metabolic pathways [58,59,60].

### 3.6. Correlation Analysis

**Figure 5:**
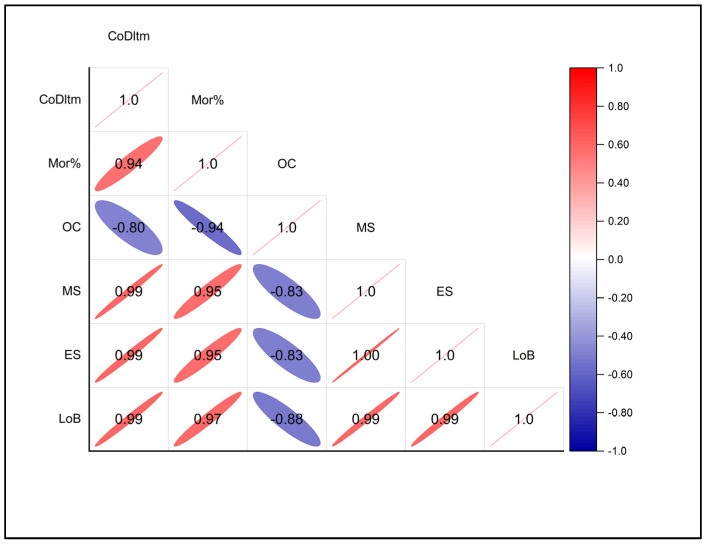
Correlation plot between deltamethrin concentration (CoDltm), mortality percentage (MOR%), and behavioral biomarkers; i.e., erratic swimming (ES), mucous secretion (MS), movement (M), erratic swimming (ES), loss of balance (LoB), and oxygen consumption (OC) for *O. mossambicus*.

According to a correlation plot for deltamethrin exposures and behavioral reactions in *O. mossambicus*, the proportion of mortality, irregular swimming, mucus secretion, and loss of balance were all positively correlated with higher deltamethrin exposures (Figure 5). Oxygen consumption and deltamethrin levels were found to be negatively correlated. Additionally, oxygen consumption revealed a negative correlation with percentage mortality, while irregular swimming, mucus secretion, and loss of balance showed positive correlations. Changes in behavior in the treated *O. mossambicus* can usually be seen as an early sign of an avoidance response caused by toxicant exposure, narcotic effects, or neurotoxic reactions. A behavioral biomarker is a designator of an early-warning signal for ecotoxicological risk assessment investigations [61,62]. The fish exposed to deltamethrin most frequently displayed loss of balance, mucus production, and irregular and abnormal swimming patterns. Neurological system impairment is probably what causes lateral swimming and loss of equilibrium [63,64]. In fish exposed to pesticides, measurements of oxygen as a surrogate for aerobic respiration and metabolic activity revealed decreased oxygen consumption because of potential inhibition of oxidative phosphorylation [65,66]. The lack of oxygen intake in the mortalities observed at high exposures is implied by the negative connection between mortality and oxygen consumption [67].

### 3.7. In Silico Read-Across Analysis

**Figure 6:**
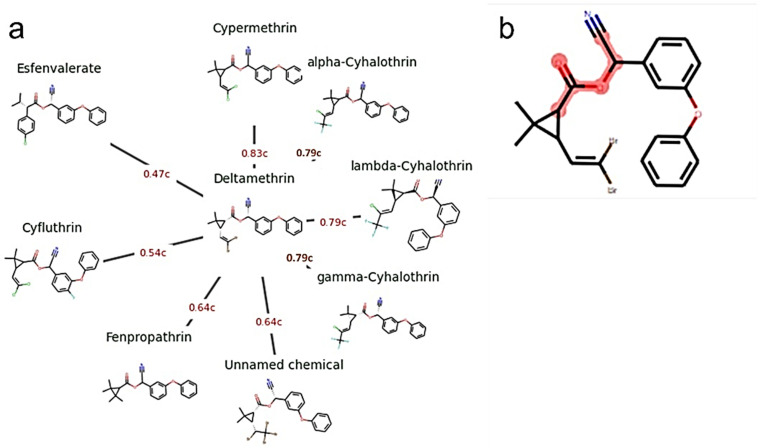
The equivalents to deltamethrin based on the Morgan fingerprints, (**a**) displays the eight closest analogues of deltamethrin. The analogues are shown in a clockwise manner and in declining order of Jaccard similarity, which is indicated by a red highlight (as decimal numbers followed by ‘c’). Where unnamed, chemical (chemical without trade name) = (S)-Cyano(3-phenoxyphenyl) methyl (1R,3S)-2,2-dimethyl-3-(1,2,2,2-tetrabromoethyl) cyclopropane-1-carboxylate. (**b**) Common substructure denoted highlighted in red shade.

On the basis of chemical and bioactivity fingerprints, a Generalized Read-Across (GenRA) analysis was carried out to identify nearby or related or analogous compounds. The redial plot in Figure 6a displays the eight substances that resemble deltamethrin the most. However, the analogy analysis based on Jaccard similarity reveals cypermethrin as the closest member of the pyrethroid family to deltamethrin, with esfenvalerate showing the least similarity; i.e., cypermethrin (0.83) > alpha-cyhalothrin (0.79) = lambda-cyhalothrin (0.79) = gamma-cyhalothrin (0.79) > fenpropathrin (0.64) > cyfluthrin (0.54) > esfenvalerate (0.47). The hazardous substructure common to the majority of the nearby compounds is depicted in Figure 6b. This hazardous substructure is a potential electrophilic agent, and molecular characteristics indicate that it can make covalent connections in terms of protein structure and DNA. This is consistent with cypermethrin affinity and inhibitory properties for enzyme proteins like choline-esterases, by interacting with the anionic substrate binding site [68]. As such, the choline esterase inhibitory properties of seven other compounds can be inferred.

#### AOP for Behavioral and Respiratory Toxicity

**Figure 7:**
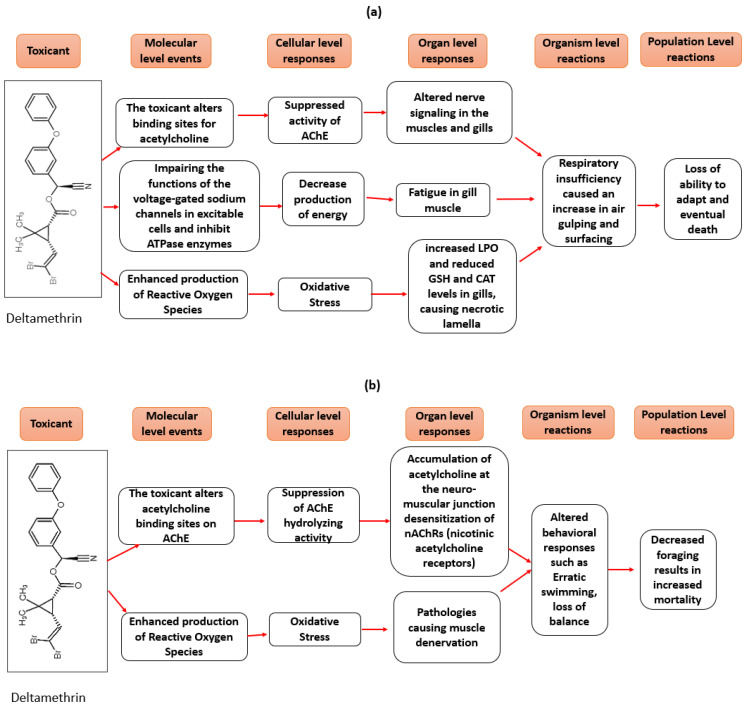
AOP for (**a**) respiratory impairment and (**b**) behavioral toxicity by deltamethrin in *O. mossambicus*. The boxes represent toxic responses documented in aquatic species, including *O. mossambicus* exposed to deltamethrin. Arrows denote postulated Key Event Relationships (KER).

The AOP framework has application in predictive and regulatory toxicology, particularly for well-studied chemicals like pyrethroids. The development of AOP based on deltamethrin-specific studies is given in Figure 7. Adverse pathways developed from toxicity endpoints in fish exposure studies reveal two plausible pathways of adverse effects on respiration and behavior; i.e., neurotoxicity via AChE inhibition and lipid peroxidation via oxidative stress (Figure 7). Developing an AOP is necessary to present the relevance of experimental data in a more structured way [69].

A major highlight for the AOP for respiratory toxicity is seen in cholinergic inhibition as a pathway to respiratory distress. In addition, exposure to contaminants may affect an organism’s physiology by altering energy acquisition and allocation [70], and its eventual ability to physically exercise respiratory organs for oxygen uptake. Onset of pathologies in respiratory tissue and the eventual compromise of tissue integrity for respiratory activity also presents a plausible pathway for manifestations of respiratory distress (Table 2). Cypermethrin, being the most similar pyrethroid to deltamethrin (Figure 7), was used to complement empirical information for the AOP.

Overall, the AOP affords visualization of pathways and identification of linkages between different biomarker responses, thus giving a rationale for predicting toxic effects across levels of biological organization [83]. In a natural ecosystem, population-level responses are most relevant for natural resource management; but, for species with special conservation status, population-level responses are less important (e.g., threatened or endangered, or highly valued to a particular stakeholder group); effects at the level of the individual may have important population consequences. The generalized AOP proposed by [83] emphasizes that adverse outcomes at the organismal level should inform the ERA (ecological risk assessment) (e.g., impaired survival, growth, reproduction). Since traditional laboratory-derived acute toxicity benchmarks (e.g., NOEC, LC_50_, and EC_50_) do not fully predict adverse effects from ecologically complex contamination scenarios [84,85,86], AOPs based on empirical toxicological endpoints will help elucidate implications for population-level effects.

## 4. Conclusions

The findings of this study using Mozambique tilapia suggest that behavior is a very sensitive endpoint, and that deltamethrin is an impactful toxin with a moderate toxic effect on *O. mossambicus*. Deltamethrin exposure generated behavioral changes that were mostly characterized by increased activity and low anxiety (reduced need for shelter), which are ecologically significant because they may enhance the danger of predation for organisms exposed to this pesticide. The progression of sublethal toxic reactions in fish exposed to deltamethrin, including increased mucus secretion, irregular swimming, increased opercular movement, and indications of uncoordinated swimming, may be a sign of cholinergic toxicity in this species. Although linkages and forecasting of responses at the level of the individual (behavioral, physiological, survival) through population (recruitment) has been demonstrated by the AOP, a more holistic picture of risks could be developed by testing a wider range of endpoints, including metabolic toxicity under acute exposure regimes. The widespread geographic occurrence of tilapias suggests that toxic responses documented for this study could be a representative scenario for a wide range of habitats.

## Figures and Tables

**Table 1:** Lethal concentrations (LC_10, 20, 50_) plus 95% fiducial limits [given in square brackets] of deltamethrin to *O. mossambicus* at 24 h–96 h exposure (ten fish per aquarium, forty fish per exposure group; four replicates).

	Lethal Concentration	Concentration with 95% Confidence Intervals (µg/L)			
		24 h	48 h	72 h	96 h
*Oreochromis mossambicus*	LC_10_	8.319 [6.442–9.347]	8.049 [6.041–9.154]	7.035 [5.658–7.914]	6.052 [4.894–6.834]
	LC_20_	9.511 [8.063–10.468]	9.406 [7.876–10.476]	7.985 [6.808–8.789]	6.839 [5.809–7.563]
	LC_30_	10.476 [9.315–11.560]	10.524 [9.288–11.853]	8.748 [7.731–9.540]	7.468 [6.547–8.169]
	LC_40_	11.377 [10.324–12.842]	11.584 [10.398–13.547]	9.457 [8.551–10.313]	8.052 [7.217–8.765]
	LC_50_	12.290 [11.174–14.411]	12.671 [11.334–15.649]	10.172 [9.310–11.193]	8.639 [7.860–9.417]

**Table 2:** Documented toxicity mechanisms of deltamethrin on fish and other animals.

sn	Compound	Endpoint	Mechanism	Reference
1	Cypermethrin	Respiratory distress	Reduction in red blood cells (RBCs) count and hemoglobin (Hb) value	[71]
2	Deltamethrin	Behavioral toxicity; Respiratory distress (increased opercular movements)	Nerve innervations in organs via acetylcholinesterase (AChE) inhibition in brain, muscle, and gills in fish	[72]
3	Deltamethrin	Neurotoxicity	Impairing the functions of the voltage-gated sodium channels in excitable cells and inhibiting ATPase enzymes	[73]
4	Cypermethrin	Neurotoxicity	Low activities of ATPases in brain of the fish exposed to cypermethrin suggests a decrease in the transmission of nerve impulse	[74]
5	Deltamethrin	Oxidative stress	Enhanced LPO while decreasing GSH and CAT levels, leading to tissue pathologies	[75]
6	Deltamethrin	Behavioral toxicity	Severe brain pathology, i.e., lesions of the telecenphalon in zebrafish correlated with increased rates of aggression behavior	[76]
7	Deltamethrin	Behavioral toxicity Oxidative stress	Behavioral inconsistencies via hematoxicity; i.e., changes in hematological parameters Induced oxidative stress (measured as an increase in ROS production, LPO, and antioxidant enzyme activities including SOD, POD, CAT, and GR), and increased metabolic enzyme activities (AAT, LDH, and GDH) in silver carp brain, gills, liver, and muscle tissues	[77]
8	Deltamethrin	Neurotoxicity	Altered binding receptor of nicotinic acetylcholine receptors in electroplax membranes of fish	[78]
9	Deltamethrin	Oxidative stress	Inhibited enzymatic activity of SOD, CAT, and GR, as well as the oxidative damage to the gills, liver, and kidney in fish.	[79]
10	Cypermethrin	Disrupted gill integrity	(a) Decreased ion levels in blood, as measured by specific ion concentrations (Na^+^, K^+^, and Cl^−^) and changes in gill Na^+^/K^+^-ATPase activity in fish	[80]
			(b) Gill pathology	[81]
11	Cypermethrin	Behavioral toxicity	Abnormal swimming behavior attributed to Ache inhibition	[82]

## Data Availability

Not applicable.
